# Magnetic Solid-Phase Extraction Based on Magnetic Sulfonated Reduced Graphene Oxide for HPLC–MS/MS Analysis of Illegal Basic Dyes in Foods

**DOI:** 10.3390/molecules26247427

**Published:** 2021-12-07

**Authors:** Shibo Cui, Xinwu Mao, Haijing Zhang, Haowei Zeng, Zihao Lin, Xuewu Zhang, Ping Qi

**Affiliations:** 1 College of Food Science and Engineering, South China University of Technology, Guangzhou 510641, China; 201920125435@mail.scut.edu.cn (S.C.); zhjcrystal1212@163.com (H.Z.); 2 Guang Zhou Institute for Food Inspection, Guangzhou 511410, China; xinwumao@163.com (X.M.); zenghw060@163.com (H.Z.); lzh050618@163.com (Z.L.); 3 Guangzhou Institute of modern Industrial Technology, Guangzhou 511458, China

**Keywords:** MSPE method, functionalized graphene oxide, HPLC–MS/MS, illegal food dyes

## Abstract

In this study, a magnetic solid-phase extraction (MSPE) method coupled with High-Performance Liquid Chromatography Mass Spectrometry (HPLC–MS/MS) for the determination of illegal basic dyes in food samples was developed and validated. This method was based on Magnetic sulfonated reduced graphene oxide (M-S-RGO), which was sensitive and selective to analytes with structure of multiaromatic rings and negatively charged ions. Several factors affecting MSPE efficiency such as pH and adsorption time were optimized. Under the optimum conditions, the calibration curves exhibited good linearity, ranging from 5 to 60 µg/g with correlation coefficients >0.9950. The limits of detection of 16 basic dyes were in the range of 0.01–0.2 µg/L. The recoveries ranged from 70% to 110% with RSD% < 10%. The results indicate that M-S-RGO is an efficient and selective adsorbent for the extraction and cleanup of basic dyes. Due to the MSPE procedures, matrix effect and interference were eliminated in the analysis of HPLC–MS/MS without the matrix-matched standards. Thus, validation data showed that the proposed MSPE–HPLC–MS/MS method was rapid, efficient, selective, and sensitive for the determination of illegal basic dyes in foods.

## 1. Introduction

As is well-known, synthetic dyes have been widely added to many foods in modern commercial food production to improve their attractiveness and to offset the variations or losses of natural colors that can occur mainly during processing or storage. Unfortunately, many of synthetic dyes were shown to be potentially neurotoxic, genotoxic, and carcinogenic additives [1]. They may pose a potential risk to human health. Therefore, most food safety authorities have established strict regulations to limit the usage of synthetic dyes in foods to protect public health. Only synthetic dyes permitted with a proved safety can be utilized in foods.

Basic dyes are a class of synthetic cationic dyes that are mainly used for textile fabric, leather, and wood product dyeing. Most basic dyes including Rhodamine B, Basic Orange 21, Basic Orange 22, and Diethyl yellow are prohibited for use in foods worldwide, and their presence in foods at any level is not permitted because these dyes can seriously endanger the health of consumers by causing allergic and asthmatic reactions, DNA damage, etc. [2]. These basic dyes have been found to illegally enhance the appearance of some food owning to the wide variety of bright colors, low cost, high stability, and better coloring properties. Consequently, it is important and necessary to develop a rapid, sensitive, and multiresidue method for the determination of the illegal use of basic dyes in foods [3].

Due to the complexity of food samples, low content of target substances, and the interference of matrix effects, it is difficult to establish a rapid, simultaneous, and sensitive analytical method for the determination of these basic dyes in food samples, especially for the development of sample pretreatment method [4]. Currently, some analytical methods such as high-performance liquid chromatography (HPLC) and liquid chromatography mass spectrometry (LC–MS) have been proposed, which require the appropriate pretreatment technology prior to analysis to remove impurities from the sample and reduce interference, improve detection sensitivity, and concentrate the target components [5]. In these methods, various sample pretreatment technologies had been developed, such as ultrasonic-assisted extraction, high-speed centrifugation, and liquid–liquid extraction [6]; solid-phase extraction [7]; and solid-phase microextraction [8,9]. However, most of the reported methods have drawbacks such as being a laborious and time-consuming procedure, use of toxic organic solvents, and low analytical efficiency of complex samples. Hence, it is necessary to develop a pretreatment technology with high extraction efficiency, short time consumption, and nontoxic solvents. Meanwhile, LC–MS methods often suffer from matrix effects, resulting in worsening of the data quality and accuracy of quantitative analyses. Therefore, an ideal sample pretreatment technology should not only integrate extraction, purification, and enrichment processes, and greatly reduce the use of organic solvents to protect human health, but also minimize the matrix effect and separate the targeted from the interfering substances with improved detection accuracy.

Recently, Magnetic solid-phase extraction (MSPE), a new derivative of SPE, has been developed for the extraction and preconcentration of a variety of organic and inorganic compounds from complex matrices. MSPE continues to receive widespread attention and application in many fields because magnetic adsorbents can efficiently separate and collect targeted substances with the use of an external magnetic field without the complex and time-consuming steps of traditional adsorption, extraction, centrifugation, and filtration. At present, MSPE combined with other analytical instruments has been widely applied for the pretreatment and enrichment of complex samples [10,11]. However, the magnetic nanoparticles (MNPs) are very crucial as a MSPE adsorbent for the efficient extraction of the analytes.

Among reported MNPs, magnetic graphene oxide (M-GO) is considered a promising iron-based nanomaterial. M-GO has a high polarization ability and tendency to π–π interactions. In addition to oxygen-containing carboxyl and hydroxyl groups [12] that convey strong hydrophilic properties, the surface of M-GO is negatively charged and, thus, can be uniformly dispersed in water or other polar solvents as a stable suspension colloid in the form of a single layer. The structure of M-GO not only provides a basis for functional modification but also increases the contact area with adsorbates, which greatly improves the adsorption performance. Therefore, it is an excellent adsorption material [13] that is a common carbon-based nanomaterial with M-GO used in MSPE. In recent years, there has been growing interest in the application of M-GO to make effective sorbents for MSPE [14,15].

Based on the above considerations, we reported a simple synthesis method for preparing a magnetic sulfonated reduced graphene oxide (M-S-RGO) as MSPE adsorbent by chemical modification [16]. The MSPE procedure was established for the selective extraction and clean-up of basic dyes and minimization of matrix effects in foods in our studies. To achieve maximum efficiency in MSPE procedures, predominant parameters, such as extraction time, pH, desorption solution (eluent), etc., were investigated. Furthermore, the adsorption mechanism was elucidated with the use of adsorption isotherms and an adsorption kinetic model. Finally, a MSPE–HPLC–MS/MS method was developed, validated, and applied to identity several commonly used, and illegal, basic synthetic food dyes in food samples under optimal conditions based on the selective adsorption effects of the M-S-RGO.

## 2. Results

### 2.1. Characterization of M-S-RGO

#### 2.1.1. Infrared Spectrum Characterization

**Figure 1:**
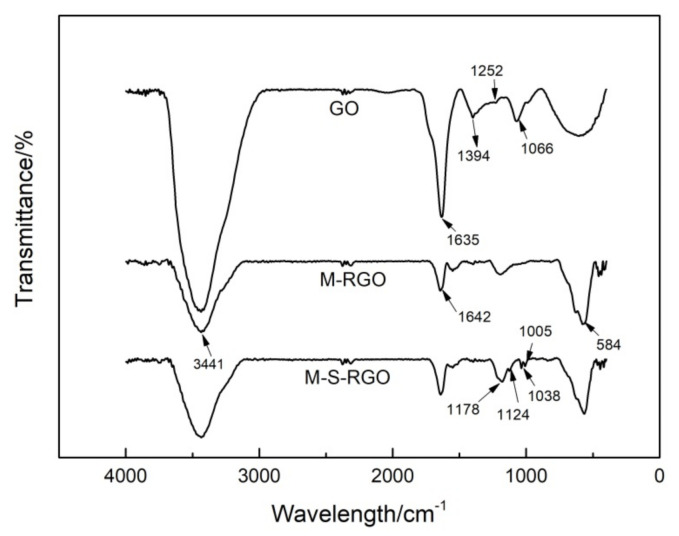
The FTIR spectrum of GO, M-RGO, and M-S-RGO.

The Fourier-transform infrared spectra of the GO, M-, and M-S-RGO are shown in Figure 1. In regard to the infrared spectrum of GO, the absorption peaks at 3441, 1727, and 1635 cm^−1^ represent the stretching vibrations of hydroxyl, carbonyl carbon–oxygen double bonds and aromatic carbon–carbon double bonds, respectively, while those at 1394, 1252, and 1066.6 cm^−1^ represent the stretching vibrations of carboxyl carbon–oxygen single bonds, ether bonds, and epoxy carbon–oxygen single bonds, respectively [17].

The infrared spectrum of the M-RGO included a Fe-O stretching vibration at 584 cm^−1^, which was absent in the GO spectrum, confirming that Fe_3_O_4_ was successfully loaded on the GO. Moreover, the absorption peaks at 1394 and 1066.6 cm^−1^ were absent from the spectrum of the M-RGO, indicating that the corresponding positions were reduced and the M-RGO was successfully prepared.

The infrared spectrum of the M-S-RGO contained three new absorption peaks at 1178, 1124, and 1038 cm^−1^, which represent the stretching vibrations of an aryl carbon–sulfur single bond and two symmetrical stretching vibrations of sulfur–oxygen double bonds, thereby confirming the successful addition of sulfonate to the prepared M-RGO.

#### 2.1.2. X-ray Photoelectron Spectroscopy (XPS) Analysis

**Figure 2:**
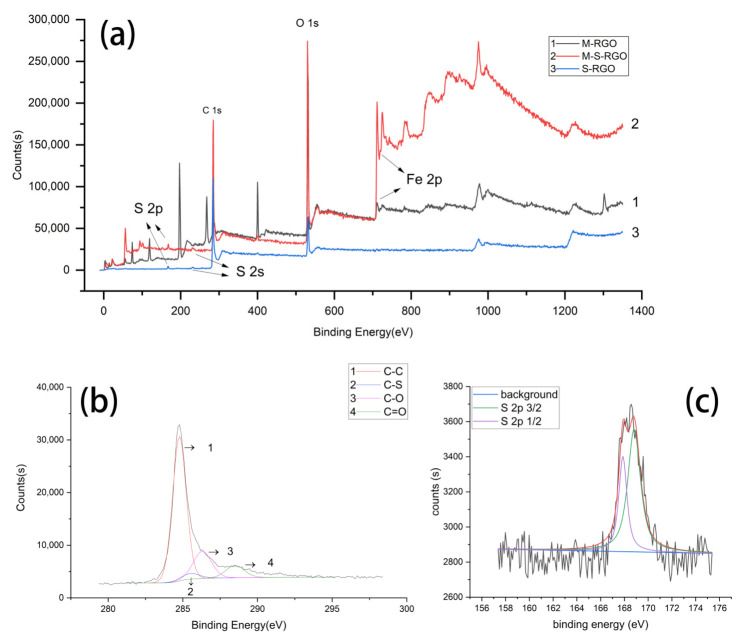
(**a**) XPS full-spectra of the M-RGO and M-S-RGO. (**b**) XPS spectrum of the C1s orbital in M-S-RGO. (**c**) XPS spectrum of the S2p orbital of the M-S-RGO.

XPS can reflect the element composition, content, molecular state, and chemical bonds of the material by measuring the electron binding energy of the inner layer and the chemical shift of an atom. The XPS spectra of the M-RGO, S-RGO, and M-S-RGO are shown in Figure 2a. The binding energies of the absorption peaks of the M-S-RGO were 231.08 and 167.08 eV, which are the characteristic absorption peaks of the S2s and S2p orbitals, respectively. Although the M-RGO contained no elemental sulfur element, the sulfur content of the M-S-RGO sample was 3.51% and the Fe content was 6.47%. As shown in Figure 2b, the C1s orbital of the M-S-RGO is divided into four peaks, corresponding to C-C (284.4 eV), C-S (285.2 eV), C-O (286.6 eV), and C=O (289.1 eV), respectively. From the peak separation results of C1s, it can be inferred that a sulfonic group was grafted onto the GO via a C–S bond. These results can be confirmed in similar research [18].

The spectrum of the S2p orbital of the M-S-RGO is shown in Figure 2c. The peak of the S2p orbital shows a perfect Gaussian distribution, indicating that the sulfur molecule had a single bond and existed only in the form of C-SO_3_H. Sulfonic acid did not combine with the material by an ester bond or any other chemical bond, indicating no possibility of a sulfonic group adsorbing on graphene. These results preliminarily confirmed that a sulfonic group was successfully grafted onto M-RGO.

#### 2.1.3. Hysteresis Curve Characterization

The hysteresis curve illustrates the ability of a material to maintain the intensity of the magnetic field after magnetization and the ability to respond to a change in the magnetic field after magnetization. 

A hysteresis curve of the M-S-RGO is shown in Figure S1. The saturation magnetic field intensity of the M-S-RGO was about 34.17 emu/g; the hysteresis curve of M-S-RGO passed through the origin and the curve had a smooth S shape, indicating that the composite material was superparamagnetic. If the saturation magnetic field intensity of the material >16.3 emu/g, the material can be separated from a mixture system with an external magnetic field [17]. The actual magnetization of the M-S-RGO was much higher than this value, so there was sufficient theoretical support for the rapid separation of this material with the use of an external magnetic field in subsequent adsorption experiments.

#### 2.1.4. Zeta Potential Characterization

The zeta potential test results of the GO and M-S-RGO are shown in Figure S2. Obviously, the zeta potentials of the GO and M-S-RGO composites were always negative, indicating a net negative charge of the surface. With an increase in pH, the potential continued to decrease, and the negative charge of the surface continued to increase. Under the same conditions, the zeta potential of the M-S-RGO is lower than that of the GO, because the aryl sulfonate radical connected to the GO further increased the negative charge of the GO surface, which also indicates higher adsorption properties of the M-S-RGO.

#### 2.1.5. Electron Microscopic Characterization

The surface structure of a material can be directly observed by SEM. SEM images of the GO and M-S-RGO composites at the same magnification are shown in Figure 2a. From the figure, GO has a two-dimensional lamellar structure with curls and folds, which can prevent accumulation of the grapheme lamellae, thereby increasing the specific surface area and improving the adsorption properties of the material while providing a large number of spatial sites for the loading of Fe_3_O_4_ nanoparticles. A SEM image of the M-S-RGO is shown in Figure 2b. From the figure, the nanoparticles are loaded on the lamellar surface of the Fe_3_O_4_ molecules, which conveys magnetism to the M-S-RGO for MSPE. After magnetic modification, the surface structure and adsorption capacity of the M-S-RGO remained unchanged.

**Figure 3:**
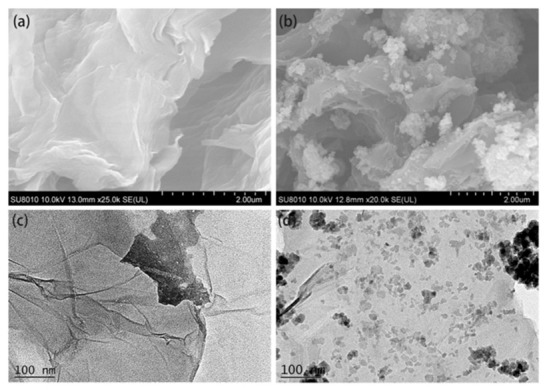
(**a**) SEM image of GO. (**b**) SEM image of the M-S-RGO. (**c**) TEM images of GO. (**d**) TEM images of the M-S-RGO.

The structure of the transparent monolayer and surface wrinkles of GO are shown in Figure 3c,d, respectively. In contrast, uniformly loaded Fe_3_O_4_ magnetic nanoparticles can be seen on the surface of the M-S-RGO and the lamellar structures of the M-S-RGO and GO are the same.

### 2.2. Optimization of MSPE Conditions

In this experiment, three typical dyes, (i.e., basic red 13, basic orange 21, basic violet 7) were chosen as targeted dyes for optimization of MSPE conditions by HPLC, which was performed with an Agilent 1260 Infinity LC System (Agilent Technologies, Inc., Santa Clara, CA, USA) equipped with Eclips Plus C18 column (4.6 mm × 150 mm; particle size 5 μm; Agilent Technologies Inc.). All three dyes were prepared at a concentration of 1 mg/mL with acetonitrile as a solvent and stored in a refrigerator. Samples prepared by M-S-RGO were analyzed by HPLC under the following conditions: column temperature, 35 °C; injection volume, 3 μL; flow rate, 1 mL/min; mobile phase (A), ultra-pure water with 0.1% formic acid; mobile phase (B), methanol; isometric elution procedure, 45% A with 55% B; equilibrium time, 10 min.

#### 2.2.1. Effect of the pH of Solution

**Figure 4:**
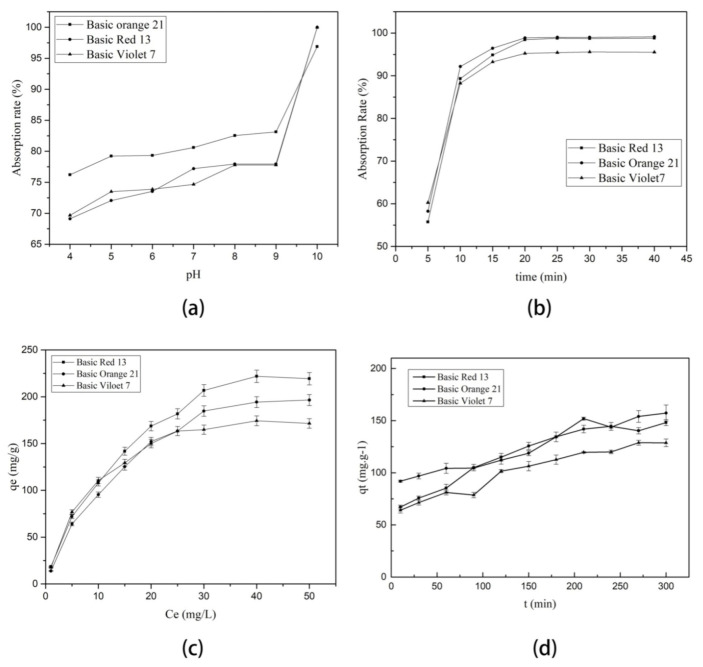
Effects of (**a**) pH and (**b**) extraction time on extraction efficiency of the M-S-RGO. (**c**) An adsorption isotherm curve of the M-S-RGO to the tested dyes. (**d**) An adsorption kinetic curve of the M-S-RGO to the tested dyes.

The influence of pH on the distribution of the surface charge of the M-S-RGO in solution plays an important role in the adsorption process. As shown in Figure 4a, at a low pH of the solution, the adsorption rate of M-S-RGO to three different dyes was only 70% or so; however, with varying pH ranging from 4 to 10, the adsorption rate increased. At a pH of about 10, the adsorption rate was almost 100%. Besides, the growth rate was initially slow and then increased from a pH of 9 to 10 and adsorption rate soared from 80% to nearly 100%.

Analysis of the adsorption mechanism of the M-S-RGO suggests that at a high pH of the solution, the surface of the M-S-RGO has a net negative charge. Basic dyes, such as basic red 13 and basic orange 21, are cationic dyes with charged surfaces. So, the electrostatic interactions between the M-S-RGO and basic dyes are very strong. Besides, there are π–π stacking interactions between the M-S-RGO and basic dyes. However, when the pH of the solution is low, the electrostatic interactions between the M-S-RGO and basic dyes are weak, leading to poorer adsorption. The experimental results showed that the best pH for M-S-RGO to adsorb basic dyes was 10; thus, follow-up experiments were conducted at pH 10.

#### 2.2.2. Effect of Adsorption Time

The adsorption of adsorbate by adsorption materials requires a certain amount of time to achieve the full utilization of the adsorption sites and to reach an adsorption equilibrium.

The effect of time on the adsorption rate of dyes is shown in Figure 4b. As indicated with time, the adsorption rate of each dye increased, which occurred rapidly at first and then slowed down and gradually reached a balance. Although the dye adsorption rate had slightly increased with time, considering the adsorption efficiency and the fact that most of the dyes are close to an adsorption equilibrium at 5–10 min, an adsorption time of 20 min was determined as sufficient for the subsequent experiments.

#### 2.2.3. Desorption and Recycle of Adsorbents

The desorption of an adsorbent with suitable eluents is an important part of the MSPE procedure for good recovery of dyes. Besides, the regeneration and reuse of adsorbents are likely to influence potential applications. A desorption method was established with 2 mg/mL of the M-S-RGO and 10 mg/L of mixed dyes (basic red 13, basic orange 21, and basic violet 7), at pH 10 and an adsorption time of 20 min. After the adsorption was completed, the solution and adsorbent were separated by the application of an external magnetic field and the supernatant was collected for HPLC analysis. In this study, desorption experiments reusing the adsorbents were performed in 5% ammonia and 5% acetic acid in acetone solutions, respectively. These processes were repeated five times. The value of cycle 0 was denoted as the adsorption of the original M-S-RGO. According to the results in Figure S5, the adsorption capacities of the dyes decreased with increasing regeneration cycle numbers. After the first regeneration cycle, the recovery efficiencies of basic orange 21, basic red 13, and basic violet 7 were 96.34%, 93.75%, and 98.06%, respectively. After the fifth cycle, the recovery efficiencies were 43.07%, 32.53%, and 74.94%. This shows that the M-S-RGO can be reused and maintains good adsorption properties after recycling.

## 3. Discussion

### 3.1. Adsorption Performance and Mechanism

#### 3.1.1. Adsorption Isotherm

An isotherm curve of the M-S-RGO for three dyes is shown in Figure 4c.

In this study, the Langmuir and Freundlich isotherm models were used to describe the equilibrium data as follows [19]:

Langmuir model: $\frac{C_{e}}{q_{e}}=\frac{1}{q\cdot K_{L}}+\frac{C_{e}}{q_{m}}$.

Freundlich model: $\log\left(q_{e}\right)=\log\left(K_{F}\right)+\left[\frac{1}{n}\right]\log\left(C_{e}\right)$.

In the Langmuir model, C_e_ (mg/L) is the dye concentration at equilibrium in the supernatant (mg/L), q_e_ is the amount of dye adsorbed by the adsorbent at equilibrium (mg/g), and K_L_ is the Langmuir isotherm constant (L/mg). In the Freundlich model, K_F_ is the Freundlich constant (mg1-nLn/g), which is an indication of the relative adsorption capacity; n is the heterogeneity factor, which represents the degree of dependence of adsorption with the equilibrium concentration; and q_m_ is the theoretical maximum adsorption capacity in a monolayer coverage of the adsorbent (mg/g).

The correlation parameters of the model fitting results are shown in Table 1. It can be concluded from Table 1 that the Langmuir equation models fitted with several dyes have a good linear correlation and the correlation coefficient of the Langmuir adsorption isotherm model is better than that of the Freundlich adsorption isotherm model, indicating monolayer adsorption of the dye by the M-S-RGO. 

#### 3.1.2. Adsorption Kinetics

An adsorption kinetic model can help to predict the adsorption rate and equilibrium time. The results of the adsorption kinetic experiments are shown in Figure 4d. As indicated in the figure, with an increase in adsorption time, the amount of the dyes adsorbed by M-S-RGO gradually increased until an adsorption equilibrium was reached. The saturated adsorption capacity of the M-S-RGO for various dyes can also be obtained from the diagram.

The collected kinetic data were analyzed using pseudo-first-order and pseudo-second-order models to arrive at the following adsorption rate formulas:

Pseudo-first-order kinetic equation:

$$\ln\left(q_{e}-q_{t}\right)=\ln\left(q_{e}\right)-K_{1}\frac{t}{2.303}$$

Pseudo-second-order kinetic equation:

$$\frac{t}{q_{t}}=\frac{1}{K_{2}q_{e}^{2}}+\frac{t}{q_{e}}$$

In the equations, q_e_ (mg/g) is the equilibrium adsorption capacity of the adsorption material, q_t_ (mg/g) is the amount of dye adsorbed by the adsorption material at each time point, K_1_ (min × 10^−1^) is the pseudo-first-order adsorption rate constant, and K_2_ (g/mg min) is the pseudo-second-order adsorption rate constant.

As indicated by the model fitting results in Table 2, the linear correlation coefficient (R^2^) of the pseudo-second-order kinetic equation model was better than that of the pseudo-first-order kinetic equation model, while the theoretical equilibrium adsorption capacity obtained by the pseudo-second-order kinetic equation model was consistent with that obtained by the actual experiment. 

The adsorption behavior of the M-S-RGO for the three dyes is in accordance with the pseudo-second-order kinetic equation model, indicating that the adsorption capacity of the M-S-RGO is controlled by the surface reaction process, a type of chemical adsorption process [20].

#### 3.1.3. The Mechanism of Dyes Adsorption

The adsorption of M-S-RGO to basic dyes is controlled by two main factors at least, π–π stacking and electrostatic attraction [21]; others, such as chemisorption, Van der Waals forces, and hydrogen bond, are secondary roles [22]. Electrostatic attraction is affected by pH. The adsorption rate of M-S-RGO to dyes kept at a low level when pH varied from 2 to 8 and then increased monotonously with increasing pH. This is because potential of M-S-RGO has changed. When the pH < 8, M-S-RGO is low negative charge, and the electrostatic attraction between M-S-RGO and basic dyes is weak. When the pH > 9, M-S-RGO is strong negative charged, and the electrostatic attraction significantly enhanced, so the adsorption rate is obviously increased. This change in potential was also verified by the characterization of zeta potential of M-S-RGO in 2.1.4. In addition, there was still adsorption of dyes when pH < 8 because the π–π stacking between M-S-RGO and dyes is unaffected by pH [23].

### 3.2. Sample Analysis

#### 3.2.1. Optimization of HPLC–MS/MS Method

In order to obtain an appropriate chromatographic response, resolution, and peak symmetries for all dyes in a reasonable time, the composition of the mobile phase and gradient elution program for HPLC was optimized. All of the selected dyes contained nitrogen groups, which easily translate to aminylium ion. HPLC–MS/MS method was performed in the polarity mode. Besides, to achieve the highest selectivity and sensitivity, the MS parameters, including the capillary voltage, cone voltage, and collision energy, were individually optimized by injecting 100 ng/mL standard solution of each compound into MS/MS with the solution of methanol and water (50:50, *v*/*v*) at a flow rate of 10 μL/min. The precursor ions and product ions for each dye were obtained in MS and MS/MS modes individually. The MS conditions of the 16 basic dyes are shown in Table S2.

#### 3.2.2. Evaluation of Matrix Effects

Despite the LC–MS/MS method becoming an essential tool for quantitative food analysis, it usually suffers from one important disadvantage of matrix effects. In order to improve the accuracy, precision, and sensitivity of a method, the matrix effect must be minimized or eliminated.

Due to the complicated ingredients of real food samples [24], the matrix effects of 16 synthetic basic dyes in selected food samples were evaluated with the MSPE procedure. Frozen grass carp, frozen yellow croaker, and tomato sauce were selected as samples in this study. HPLC–MS/MS analysis of the samples was performed with the detection method described in Section 4.2. As shown in the detection results of the dye substrate effects (Figure S3), with some exceptions, most of the dyes had no obvious matrix effect in these selected food samples, as the error due to the matrix effect was within ±20%, which indicates that the MSPE procedure based on M-S-RGO can reduce the matrix effect of dyes in the HPLC–MS/MS analysis. Owing to the elimination of matrix effects, the developed MSPE–HPLC–MS/MS method avoided the laborious and time-consuming processes without the use of matrix-matched standards.

#### 3.2.3. Method Validation and Application in Real Food Samples

In order to validate the potential application of the proposed method for the detection of synthetic basic dyes, the performance of the MSPE–HPLC–MS/MS method was evaluated under optimum conditions.

The calibration curves, linear range of the tested dyes with a regression equation, limit of detection (S/N = 3) [25], and correlation coefficient are shown in Table S3. According to the data presented in Table S3, a satisfactory linearity was obtained for these basic dyes in a range from 5 to 60 µg/L. The correlation coefficients among the curves of each dye were greater than 0.995, indicating good linearity of the dyes and that the detection method was both stable and reliable. The limit of detection of all basic dyes was less than 0.2 (µg/L), demonstrating high sensitivity of the detection method.

In this study, frozen grass carp, frozen yellow croaker, and tomato sauce were selected as spiked samples. According to a previously reported method, the experiments were conducted under optimal conditions with spiked amounts of 0.01 µg/g, 0.05 µg/g, and 0.1 µg/g. As shown by the results presented in Table 3, the recoveries ranged from 75% to 110% for most dyes, which met the requirement of analytical detection. Meanwhile, according to the data in Table 3, as the spiked levels increased, the recovery of each dye also increased, possibly because the absorptive interaction between the M-S-RGO and the basic dyes was stronger than that of the desorption interaction. The relative standard deviation of each dye was less than 10%, indicating better reproducibility and high accuracy of this method.

## 4. Materials and Methods

### 4.1. Reagents and Materials

References standards of basic green 1, rhodamine B, rhodamine 6G, crystal violet, basic violet 1, and basic blue 7 were purchased from Dr. Ehrenstorfer GmbH (Augsburg, Germany). Basic orange 21, ethyl violet 4, basic red 46, basic red 13, and basic red 14 were acquired from Sigma-Aldrich Productions GmbH (Steinheim am Albuch, Germany). Basic blue 26, basic orange 22, basic blue 11, and basic violet 7 were procured from Tokyo Chemical Industry Co., Ltd. (Tokyo, Japan). HPLC-grade acetonitrile and methanol were obtained from Thermo Fisher Scientific (Pittsburgh, PA, USA). All chemicals used were of analytical grade. FeCl_3_·6H_2_O, FeCl_2_·4H_2_O, NaHCO_3_, NaOH, NaBH_4_, NaNO_2_, and p-aminobenzenesulfonic acid were purchased from Tianjin Fuchen Chemicals Reagent Factory (Tianjin, China). Formic acid, 36% HCl, and 24% NH_3_·H_2_O were acquired from Guangzhou Chemical Reagent Factory (Zhaoqing, Guangdong province, China). GO was obtained from Nanoinnova Technologies SL (Madrid, Spain).

### 4.2. Instrumentation

The infrared spectrum of M-S-RGO was obtained with a NicoletTM iSTM 10 Fourier-transform infrared spectrometer (Thermo Fisher Scientific, Waltham, MA, USA). The element and valence states of the elements on the surface of the M-S-RGO were qualitatively and quantitatively analyzed with a K-AlphaTM X-ray photoelectronic spectrometer (Thermo Fisher Scientific). The magnetic field of the M-S-RGO was detected with a vibrating sample magnetometer (VSM 7404 Lake Shore Cryotronics, Inc., Columbus, OH, USA). The Zeta potential of the M-S-RGO was analyzed with a Zetasizer Nano ZS90 nanoparticle size analyzer (Malvern Panalytical Ltd., Malvern, UK). The morphology of the M-S-RGO was analyzed with an ultra-high-resolution scanning electron microscope (SEM) (SU-8010, Hitachi Corporation, Tokyo, Japan), and the structure was observed with a transmission electron microscope (TEM) (H7650 Hitachi Corporation).

HPLC–MS/MS analysis was performed using an Acquity I-Class UPLC system (Waters Corporation, Milford, MA, USA) coupled with a Xevo TQ-S Triple Quadrupole Mass Spectrometer equipped with an electrospray ionization source (Waters Corporation), and an Acquity BEH C18 column (2.1 mm × 100 mm; particle size,1.7 μm; Waters Corporation) was used for HPLC–MS/MS analysis. The mobile phase consisted of solvent A (89.9% water with 10% acetonitrile and 0.1% formic acid) and solvent B (99.9% acetonitrile with 0.1% formic acid). All separations were performed at a temperature of 40 °C and flow rate of 0.4 mL/min with the following optimized gradient 0–2.5 min, 0–30% B; 2.5–4.7 min, 30% B; 4.7–7 min, 30–95% B; 7–8 min, 95% B; 8–12 min, 95–0% B; 0–2.5 min, 0–30% B; 2.5–4.7 min, 30% B; 4.7–7 min, 30–95% B; 7–8 min, 95% B; and 8–12 min, 95–0% B. Then, the gradient was returned to the original proportion within 1 min and maintained for 1 min to ensure baseline stability for the next injection. The injection volume was 2 μL. ESI–MS/MS spectra were acquired via the positive polarity switching mode with MS/MS detection and multiple reaction monitoring of the two most intense daughter ions of each dye. Analysis of the basic dyes was conducted in the positive mode with a capillary voltage of 2.50 kV, source temperature of 150 °C, desolvation temperature of 500 °C, cone gas flow of 1000 L/h, and desolvation gas flow of 150 L/h. Nitrogen was used as the nebulizing and desolvation gas; argon used as the collision gas at a flow rate of 0.25 mL/min. Quantification was based on the external standard method. A best fit standard curve was prepared by linear regression of the peak areas versus concentration.

### 4.3. Preparation of M-S-RGO

The M-GO was prepared by the coprecipitation method [26]. Briefly, 150 mg of GO was dispersed in 150 mL of ultrapure water by ultrasonication for 40 min. A total 2.3 g of FeCl_3_·6H_2_O and 0.85 g of FeCl_2_·4H_2_O were successively dissolved in 30 mL of ultrapure water, then stirred in a nitrogen-protected environment at 60 °C. Following the addition of the GO dispersion, the pH of the solution was adjusted to about 9–10 with ammonia, then stirred at 60 °C for 4 h. The M-GO was isolated and washed with ultrapure water and dried in an oven at 60 °C. M-RGO was prepared by dispersing 150 mg of M-GO in 150 mL of ultrapure water by ultrasonication for 40 min. Then, 0.6 g of NaBH_4_ dissolved in 15 mL of ultrapure water was slowly added to the M-GO dispersion while stirring. The pH of the dispersion was adjusted to 9.0–9.3 with 5% NaHCO_3_ solution at 80 °C for 3 h. The M-RGO was isolated, washed with ultra-pure water, and then redispersed in 150 mL of ultrapure water and stored in a refrigerator at 5 °C.

The M-S-RGO was prepared by dissolving 0.5 g p-aminobenzenesulfonic acid in 5 mL of NaOH (2%) in a beaker. Then, 0.2 g NaNO_2_ was added, and the solution was shaken until the color turned orange. Afterward, 10 mL of ultrapure water cooled to 5 °C was added to the solution along with 1.2 mL of concentrated hydrochloric acid (36%), while stirring about 30 min at 5 °C with ice bath. This diazonium salt suspension was quickly added to the M-RGO dispersion, which had been precooled to 5 °C prior, then stirred continuously in an ice bath at 5 °C for 8 h. Afterward, the products were separated, washed with ultrapure water, and dried in an oven at 60 °C.

### 4.4. Establishment of an Adsorption Isotherm Experiment

An adsorption isotherm experiment to reveal the adsorption mechanism of the M-S-RGO was established by fitting the adsorption experimental data with a specific adsorption equation model. The experiment procedure is as follows: 1 mg/mL of M-S-RGO and a mixture of dyes were prepared. Groups containing different concentrations of dyes were set up. Other factors were kept exactly the same for each group. When the experiment started, the mixed solution was shaken for more than 30 min. The adsorption materials were then separated with strong magnets. The supernatant was collected for the analysis of HPLC–MS/MS.

### 4.5. Establishment of the Adsorption Kinetics Experiment

At 298 K, 5 mg/mL of the M-S-RGO was prepared and an appropriate amount was added to the mixed dye solution (100 mg/L). The mixture was then shaken. Every 30 min, an aliquot of the mixed solution was collected, the adsorption material was separated with the use of a strong magnetic field, and the supernatant was collected for the analysis of HPLC–MS/MS.

### 4.6. Food Samples

All of the food samples, including frozen yellow croaker, frozen grass carp, and tomato paste were purchased from the local common market in Guangzhou (China). Samples were mixed homogeneously in a high-speed food blender and stored at −20 °C until analysis. For the spiked samples, specific volumes of standard solutions of mixed basic dyes were added to the blank tomato samples at 0.01, 0.05, and 0.1 μg/g, and then prepared as aforesaid.

### 4.7. MSPE Procedure

A total 2.0 g of homogenized sample was accurately weighed, placed in a 50 mL plastic centrifuge tube with conical bottom, and then shaken for 30 min with 10 mL of methanol. Afterward, the sample was centrifuged at 8000 r/min for 10 min. Following collection of the supernatant, 10 mL of the methanol was added to the tube and the process was repeated. All of these resulting supernatants were collected and dried to about 1 mL under a gentle nitrogen stream. Then, 2 mL of the M-S-RGO (5 mg/mL) was added. The solution was diluted to 10 mL with water (pH 10) and then shaken for 30 min to ensure that the dyes fully absorbed. Afterward, the M-S-RGO was separated with the use of an external magnetic field and desorbed with acetone containing 5% ammonia. Then, the desorption procedure was repeated using acetone with 5% acetic acid. The pooled solution was dried to about 1 mL under a gentle nitrogen stream, and diluted to 10 mL with methanol for the analysis of HPLC–MS/MS.

## 5. Conclusions

In this study, a novel MSPE adsorbent (M-S-RGO) material was developed and successfully applied for the pretreatment concentration and purification of food samples. Simultaneously, HPLC–MS/MS was used for the detection of 16 synthetic basic dyes in food samples. This simple, rapid, and green method overcomes a series of problems in traditional extraction procedures. Thanks to the use of the M-S-RGO for sample pretreatment, toxic organic solvents were reduced for the extraction procedure and the time-consuming filtration and centrifugation procedures were not needed. For the detection of synthetic basic dyes in food samples, no obvious matrix effect was observed. Finally, under optimal conditions, the M-S-RGO used in the pretreatment process was coupled with HPLC–MS/MS for the detection of 16 kinds of basic dyes in food samples. Compared with some MSPE methods that have been reported, this method can be applied to detection of a wider range of synthetic basic dyes with lower LODs (Table 4). The results demonstrated that MSPE based on M-S-RGO as adsorbents together with HPLC–MS/MS is an efficient, selective, and sensitive method for the routine analysis of synthetic basic dyes in food samples and might also have applications with biochemical and environmental samples.

## Figures and Tables

**Table 1:** Parameters of adsorption isotherm of the M-S-RGO.

Dye	Langmuir Model			Freundlich Model		
	K_L_	q_m_	R^2^	K_F_	1/n	R^2^
Basic violet 7	1.10	213.22	0.9915	24.26	0.56	0.9160
Basic red 13	0.63	300.30	0.9878	22.66	0.63	0.9667
Basic orange 21	0.59	272.48	0.9893	17.91	0.67	0.9560

**Table 2:** Parameters of kinetic model of the M-S-RGO.

Dye	Experiment q_e_	Quasi-First-Order Kinetic			Quasi-Second-Order Kinetic		
		K_1_	Theoretical q_e_	R^2^	K_2_	Theoretical q_e_	R^2^
Basic violet 7	128.34	1.84	83.86	0.9465	0.18	141.84	0.9727
Basic red 13	144.60	3.66	167.71	0.6180	0.18	141.84	0.9727
Basic orange 21	155.00	2.58	127.67	0.7037	0.16	168.35	0.9638

**Table 3:** Recovery and RSD of the analytes in different matrices.

Dyes	Spiked Level µg·g^−1^	Recovery% (RSD %)		
		Tomato Sauce	Yellow Croaker	Grass Carp
Basic Orange 21	0.01	88.5 (3.5)	76.4 (5.1)	71.4 (4.8)
	0.05	92.4 (3.2)	78.2 (5.1)	76.9 (5.1)
	0.10	95.2 (3.4)	82.3 (4.8)	79.1 (4.9)
Basic Red 46	0.01	81.3 (4.9)	87.3 (3.7)	82.4 (4.6)
	0.05	85.2 (5.1)	89.2 (4.6)	81.1 (4.5)
	0.10	89.3 (5.0)	93.1 (3.9)	86.4 (4.5)
Basic Violet 1	0.01	93.2 (3.4)	87.2 (4.4)	88.7 (4.1)
	0.05	94.9 (2.9)	89.6 (4.3)	90.6 (3.8)
	0.10	97.3 (3.3)	94.3 (3.3)	92.5 (4.3)
Crystal Violet	0.01	94.4 (3.5)	75.4 (4.7)	90.3 (3.9)
	0.05	97.5 (3.1)	77.2 (4.8)	92.7 (3.4)
	0.10	100.9 (3.3)	85.1 (4.0)	96.4 (3.7)
Basic Green 1	0.01	92.1 (4.1)	67.4 (4.8)	60.6 (4.7)
	0.05	96.3 (3.2)	70.7 (4.6)	63.7 (4.6)
	0.10	95.1 (3.8)	69.4 (4.4)	67.9 (4.9)
Basic Orange 22	0.01	91.9 (4.2)	66.3 (4.8)	76.9 (4.8)
	0.05	93.0 (4.1)	66.9 (5.1)	73.8 (5.3)
	0.10	94.3 (4.0)	67.9 (4.7)	70.4 (4.4)
Basic Blue 11	0.01	97.9 (4.8)	82.2 (4.5)	88.4 (3.9)
	0.05	102.4 (5.5)	75.5 (4.5)	91.3 (3.7)
	0.10	104.1 (4.2)	90.2 (4.2)	93.5 (4.2)
Rhodamine 6G	0.01	88.4 (4.4)	78.7 (4.5)	75.3 (4.8)
	0.05	87.9 (4.3)	80.8 (4.3)	78.7 (5.4)
	0.10	91.2 (4.9)	84.4 (5.1)	77.3 (4.3)
Rhodamine B	0.01	84.2 (4.7)	89.3 (4.7)	89.7 (3.9)
	0.05	83.9 (4.3)	87.6 (4.2)	91.1 (3.6)
	0.10	87.3 (4.2)	95.3 (3.2)	97.4 (3.5)
Ethyl Violet 4	0.01	98.6 (4.9)	90.9 (4.2)	82.5 (4.7)
	0.05	109.1 (5.2)	89.3 (3.9)	78.6 (3.9)
	0.10	105.5 (4.3)	93.9 (3.5)	88.2 (4.3)
Basic Blue 7	0.01	94.3 (3.5)	85.6 (4.2)	72.5 (4.8)
	0.05	97.7 (3.3)	85.3 (4.6)	75.9 (4.2)
	0.10	103.1 (3.7)	87.6 (4.3)	73.4 (5.4)
Basic Red 14	0.01	84.7 (4.2)	83.5 (4.1)	84.6 (4.3)
	0.05	83.0 (3.5)	77.3 (3.8)	87.3 (4.1)
	0.10	89.8 (4.4)	89.7 (3.9)	92.8 (4.0)
Basic Red 13	0.01	87.4 (4.1)	68.6 (4.5)	88.1 (3.6)
	0.05	89.0 (3.5)	70.4 (4.6)	91.0 (3.4)
	0.10	93.7 (3.8)	87.4 (4.5)	97.8 (3.4)
Basic Violet 7	0.01	94.2 (3.9)	79.5 (4.3)	84.6 (3.8)
	0.05	98.71 (3.3)	80.7 (4.8)	88.8 (3.5)
	0.10	101.4 (4.7)	92.4 (3.8)	96.4 (3.3)
Basic Blue 26	0.01	92.6 (3.4)	92.8 (3.5)	91.5 (3.4)
	0.05	94.9 (3.2)	94.4 (3.6)	95.9 (3.2)
	0.10	96.9 (3.9)	95.6 (3.2)	94.4 (3.2)
Malachite Green	0.01	92.7 (3.6)	79.4 (3.9)	77.6 (4.0)
	0.05	98.2 (3.2)	82.5 (4.5)	75.9 (4.3)
	0.10	104.5 (4.5)	90.3 (3.7)	87.3 (3.9)

**Table 4:** Comparison with other MSPE-proposed methods for detection of dyes.

Analyte	Method	Adsorbent	LOD (µg/L)	Ref.
Methyl Red, Methyl Orange	MSPE–HPLC–MS/MS	MHNTs	0.05	[20]
Methyl Red, Methyl Orange	MSPE–HPLC	MHNTs@C16mimBr	0.12	[27]
Sudan Dyes, Para Red	MSPE–HPLC	cMWCNT-γ-Fe_2_O_3_	0.31	[28]
Cationic Dyes	SPE–HPLC	Nanofibers	0.3–0.5	[29]
16 Basic Dyes	MSPE–HPLC–MS/MS	M-S-RGO	0.01–0.2	This work

## Data Availability

Data is contained within the article.

## Supplementary Materials

The following is available in the Supplementary material: Table S1: Properties of illegal dyes used in the study: NO. of dyes, Dye Name, CAS, Formula, RT (min), MW (g/mol) and Structure; Table S2: Mass spectrometry detection method of 16 basic dyes; Table S3: Calibration curve and limit characteristics of 16 basic dyes; Table S4: MSPE Comparation of M-GO, M-RGO and M-S-RGO; Figure S1: The magnetic hysteresis loop of M-S-RGO; Figure S2: The Zeta potential images of GO and M-S-RGO; Figure S3: The matrix effect of 16 synthetic basic dyes in 3 samples; Figure S4: Reusability of M-S-RGO in the remove of 3 synthetic basic dyes; Figure S5: Schematic illustration of the M-RGO sulfonation process; Figure S6: HPLC-MS/MS chromatograms of 16 synthetic basic dyes.

## Sample Availability

Samples of the compounds are available from the authors.
