# Supplementary material for: Magnetic Solid-Phase Extraction Based on Magnetic Sulfonated Reduced Graphene Oxide for HPLC–MS/MS Analysis of Illegal Basic Dyes in Foods
Source: Molecules. 2021 Dec 7;26(24):7427. doi: 10.3390/molecules26247427 (PMC8708935; doi:10.3390/molecules26247427)
Supplement: Supplementary file 1 [file molecules-26-07427-s001.zip › molecules-1461025supp.pdf]

Supplementary Material

Table S1. Properties of illegal dyes used in the study: NO. of dyes, Dye Name, CAS, Formula, RT (min), MW (g/mol) and Structure.

No.	Dye Name	CAS	Formula	RT (min)	MW (g/mol)	Molecular Structure
1	Basic Orange 21	3056-93-7	$C_{22}H_{23}ClN_2$	6.48	350.88	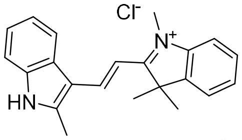
2	Basic red 46	12221-69-1	$C_{18}H_{21}N_6{\cdot}Br$	3.37	403.319	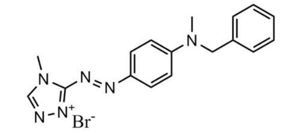
3	Basic Violet 1	8004-87-3	$C_{24}H_{28}ClN_3$	7.26	393.95	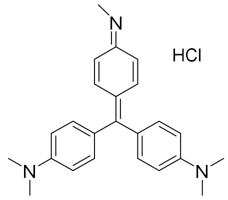
4	Crystal Violet	548-62-9	$C_{25}H_{30}ClN_3$	7.53	407.99	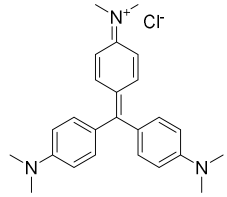
5	Basic Green 1	633-03-4	$C_{27}H_{34}N_2O_4S$	7.93	482.63	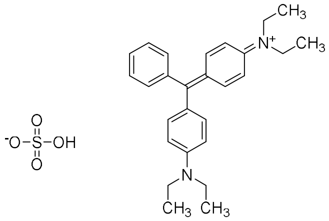
6	Basic Orange 22	4657-00-5	$C_{28}H_{27}ClN_2$	7.37	426.98	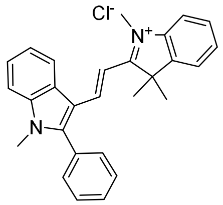
7	Basic Blue 11	2185-86-6	$C_{29}H_{32}N_3Cl$	7.72	458.05	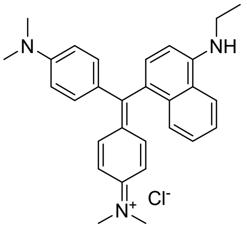
8	Rhodamine 6G	989-38-8	$C_{28}H_{31}ClN_2O_3$	7.43	479.01	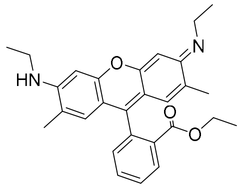

No.	Dye Name	CAS	Formula	RT (min)	MW (g/mol)	Molecular Structure
9	Rhodamine B	81-88-9	$C_{28}H_{31}ClN_2O_3$	6.95	479.01	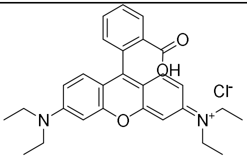
10	Ethyl Violet 4	2390-59-2	$C_{31}H_{42}ClN_3$	9.27	492.14	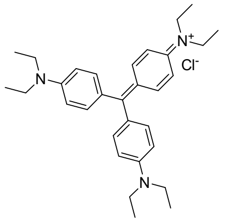
11	Basic Blue 7	2390-60-5	$C_{33}H_{40}N_3Cl$	8.76	514.15	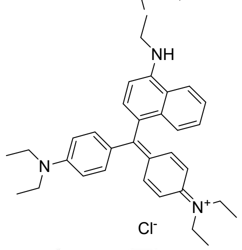
12	Basic red 14	12217-48-0	$C_{23}H_{26}N_3Cl$	3.44	379.926	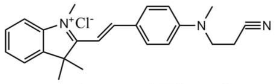
13	Basic red 13	3648-36-0	$C_{22}H_{26}N_2Cl_2$	4.95	389.4	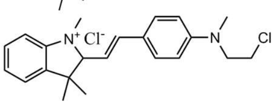
14	Basic Violet 7	6441-82-3	$C_{24}H_{30}Cl_2N_2$	7.38	417.41	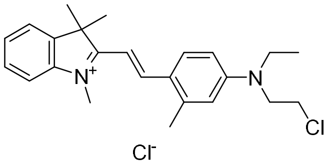
15	Basic Blue 26	2580-56-5	$C_{33}H_{32}N_3Cl$	7.87	506.09	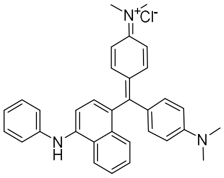
16	Malachite green	13425-25-7	$C_{23}H_{25}N_2$ Cl	5.17	----	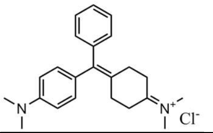

Table S2. Mass spectrometry detection method of 16 basic dyes.

Dyes	Retention time/min	Parent(m/z)	Daughter(m/z)		Cone(V)	Collision(V)	
**Basic Orange 21**	**3.67±0.24**	315.0	285.0	300.0*	10	24	30
Basic Red 46	3.37±0.23	321.4	90.9	196.3*	15	30	15
Basic Violet 1	5.90±0.195	358.8	327.3	343.3*	15	50	35
Crystal Violet	6.16±0.205	372.3	340.3	356.1*	15	55	38
Basic Green 1	6.44±0.16	385.0	297.0	341.0*	10	50	38
Basic Orange 22	6.05±0.145	391.0	361.0	376.0*	10	35	28
Basic Blue 11	6.34±0.16	422.5	272.5	393.3*	15	43	32
Rhodamine 6G	6.11±0.185	443.0	341.0	415.0*	10	35	48
Rhodamine B	6.07±0.105	443.0	355.0	399.0*	10	60	45
Ethyl Violet 4	6.84±0.14	456.4	368.1	412.1*	15	55	45
Basic Blue 7	6.74±0.155	478.7	389.9	434.2*	15	55	43
Basic Red 14	3.44±0.155	344.2	274.0	289.0*	15	35	30
Basic Red 13	4.95±0.155	353.5	289.0*	338.0	15	33	24
Basic Violet 7	6.07±0.15	381.0	317.0	366.0*	10	32	25
Basic Blue 26	6.45±0.15	470.3	349.2	454.0*	15	37	43
Malachite Green	5.17±0.195	329.0	208.0*	313.0	10	35	35

* Quantitative ion.

Table S3. Calibration curve and limit characteristics of 16 basic dyes.

Num	Dyes	Regression equation	Linear range ($\mu g \cdot L^{-1}$)	LOD ($\mu g \cdot L^{-1}$)	Correlation coefficient
1	Basic Orange 21	Y=41039 X-2553	5-60	0.0842	0.9980
2	Basic Red 46	Y=17675X+1829	5-60	0.0475	0.9978
3	Basic Violet 1	Y=5165X-3856	5-60	0.1236	0.9995
4	Crystal Violet	Y=86491X+92025	5-60	0.0324	0.9975
5	Basic Green 1	Y=76057 X+16635	5-60	0.0197	0.9989
6	Basic Orange 22	Y=55214X-178	5-60	0.0198	0.9977
7	Basic Blue 11	Y=13825X+9367	5-60	0.0314	0.9989
8	Rhodamine 6G	Y=56373X+24679	5-60	0.0194	0.9958
9	Rhodamine B	Y=54159X+39213	5-60	0.0317	0.9962
10	Ethyl Violet 4	Y=58032X+41012	5-60	0.0374	0.9939
11	Basic Blue 7	Y=40522X+45623	5-60	0.0253	0.9959
12	Basic Red 14	Y=18534X-9172	5-60	0.0564	0.9987
13	Basic Red 13	Y=2640X-2053	5-60	0.0441	0.9988
14	Basic Violet 7	Y=5569X+1958	5-60	0.0151	0.9952
15	Basic Blue 26	Y=12828X+4001	5-60	0.0799	0.9955
16	Malachite Green	Y=10897X-3388	5-60	0.1885	0.9975

Table S4. MSPE Comparation of M-GO, M-RGO and M-S-RGO.

	M-GO	M-RGO	M-S-RGO
Basic Orange 21	11.77 (4.64)	13.62 (4.97)	96.94 (3.92)
Basic Red 46	16.52 (4.81)	15.60 (5.07)	97.52 (4.84)
Basic Violet 1	13.70 (5.33)	18.42 (4.72)	97.13 (4.79)
Crystal Violet	12.42 (4.36)	9.09 (4.43)	95.84 (5.20)
Basic Green 1	17.83 (4.81)	17.49 (4.13)	96.49 (4.63)
Basic Orange 22	16.24 (5.55)	22.08 (5.63)	98.20 (4.87)
Basic Blue 11	18.72 (4.34)	20.26 (4.90)	96.85 (5.24)
Rhodamine 6G	6.18 (4.19)	12.79 (4.69)	97.31 (4.98)
Rhodamine B	9.92 (4.98)	15.24 (5.07)	97.24 (4.35)
Ethyl Violet 4	16.87 (5.02)	26.13 (4.96)	95.42 (4.61)
Basic Blue 7	15.07 (3.77)	22.40 (4.28)	97.07 (5.38)
Basic Red 14	18.61 (4.48)	16.55 (4.75)	96.62 (4.92)
Basic Red 13	14.13 (5.07)	26.17 (4.36)	97.43 (4.71)
Basic Violet 7	10.41 (4.62)	13.38 (5.18)	96.58 (4.23)
Basic Blue 26	14.33 (5.37)	19.65 (4.62)	97.60 (4.46)
Malachite Green	7.87 (5.44)	8.32 (4.83)	94.71 (4.89)

*All dyes were 1mg/L, M-GO, M-RGO and M-S-RGO were 1mg/mL, n=3.

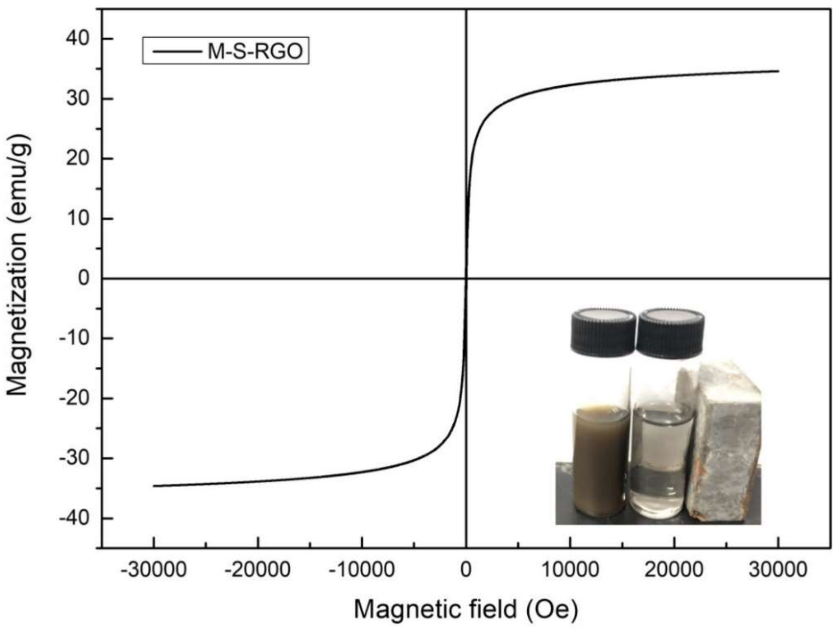

Figure S1. The magnetic hysteresis loop of M-S-RGO.

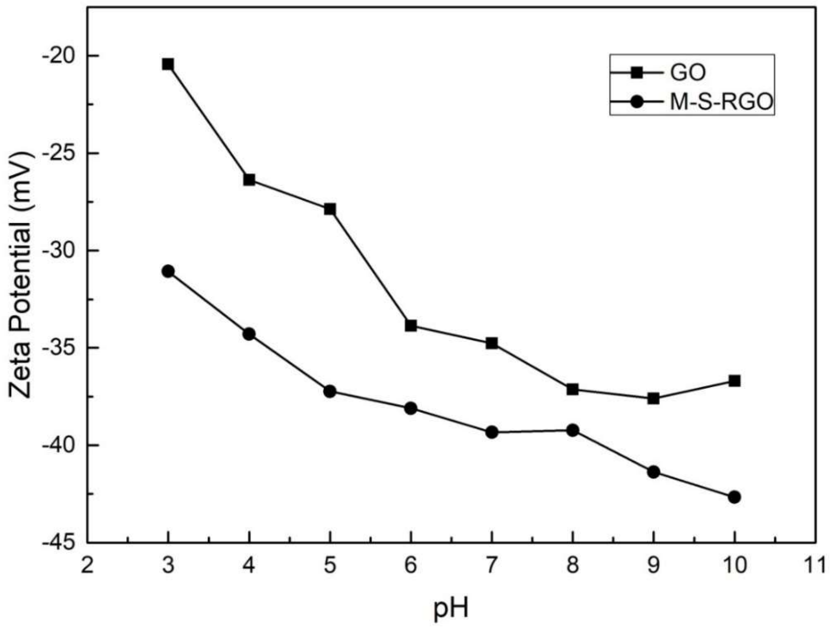

Figure S2. The Zeta potential images of GO and M-S-RGO.

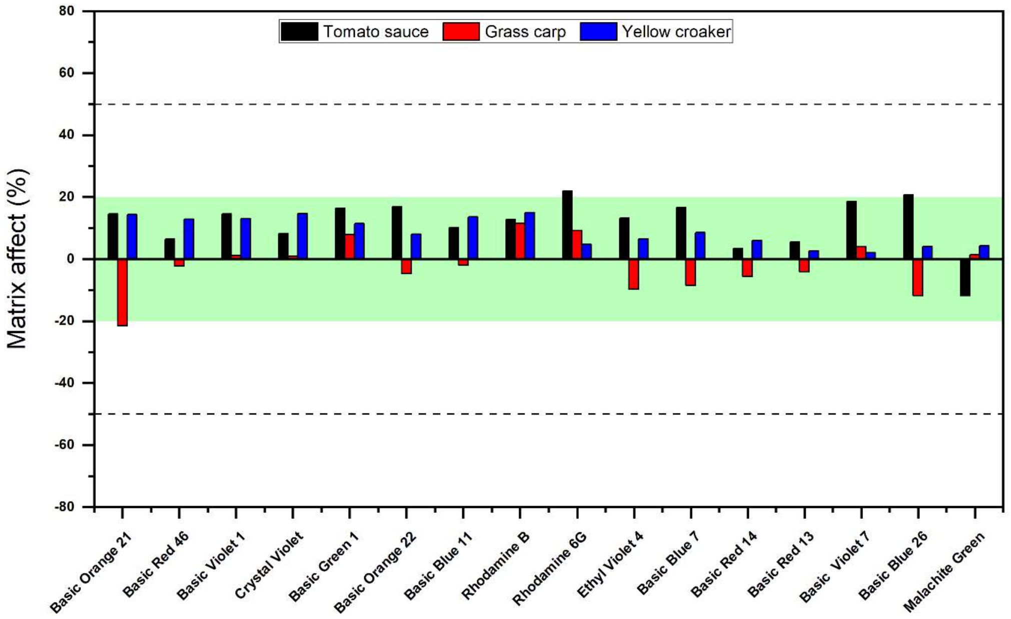

Figure S3. The matrix effect of 16 synthetic basic dyes in 3 samples.

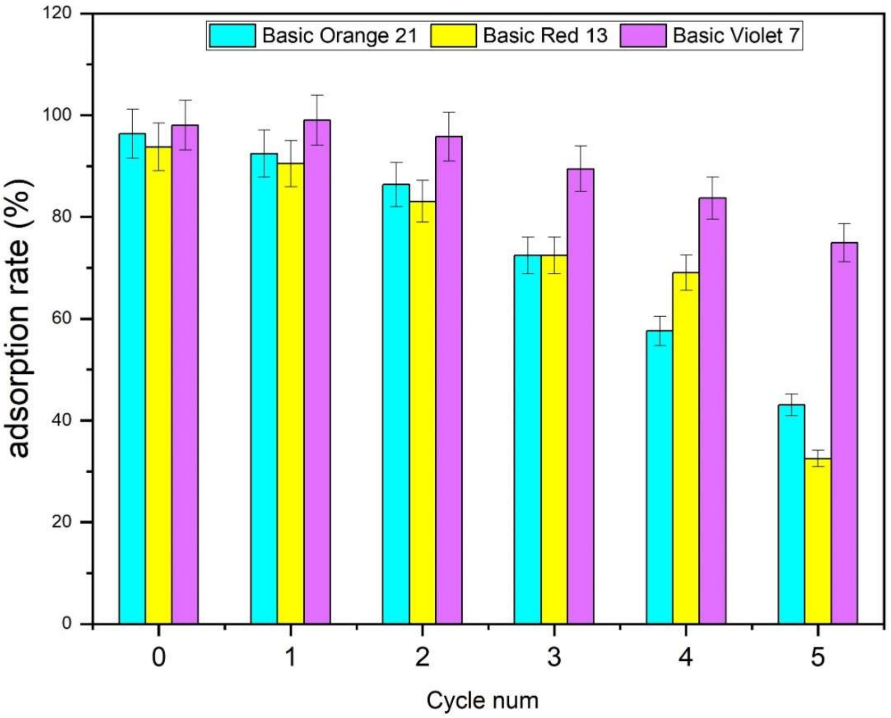

Figure S4. Reusability of M-S-RGO in the remove of 3 synthetic basic dyes.

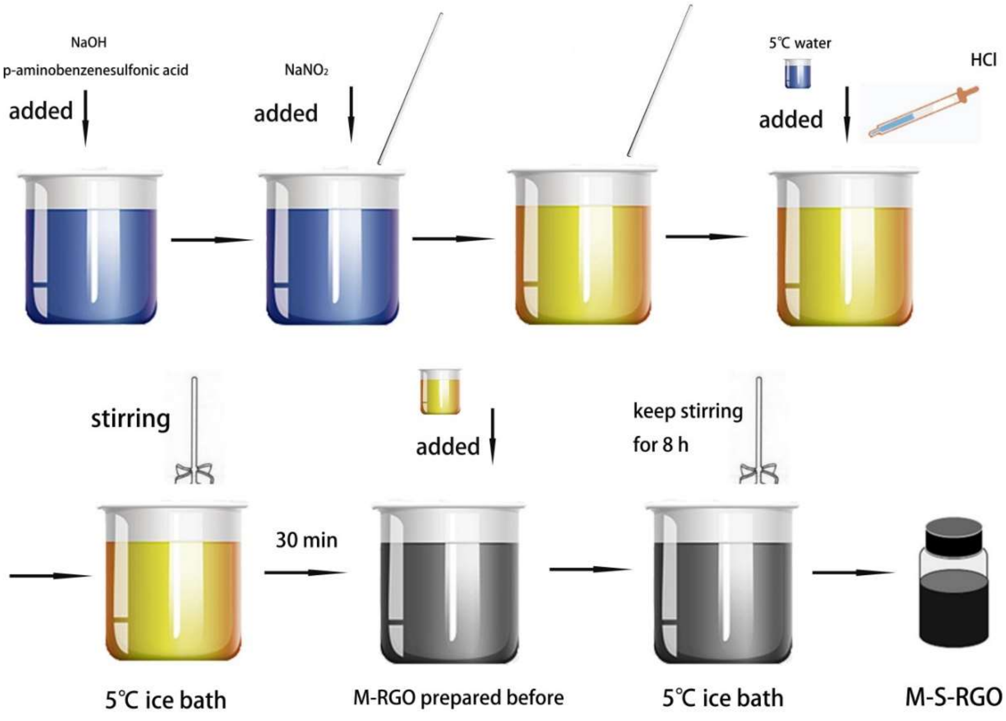

Figure S5. Schematic illustration of the M-RGO sulfonation process.

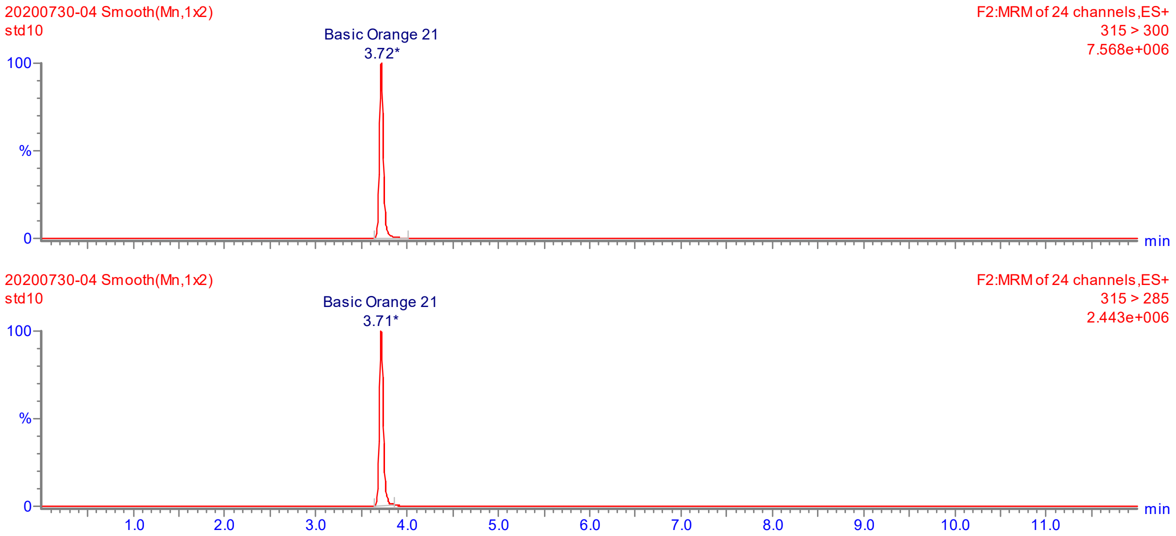
20200730-04 Smooth(Mn,1x2)
std10
Basic Orange 21
3.72*
F2:MRM of 24 channels,ES+
315 > 300
7.568e+006
100
%
0
min
20200730-04 Smooth(Mn,1x2)
std10
Basic Orange 21
3.71*
F2:MRM of 24 channels,ES+
315 > 285
2.443e+006
100
%
0
min
1.0
2.0
3.0
4.0
5.0
6.0
7.0
8.0
9.0
10.0
11.0

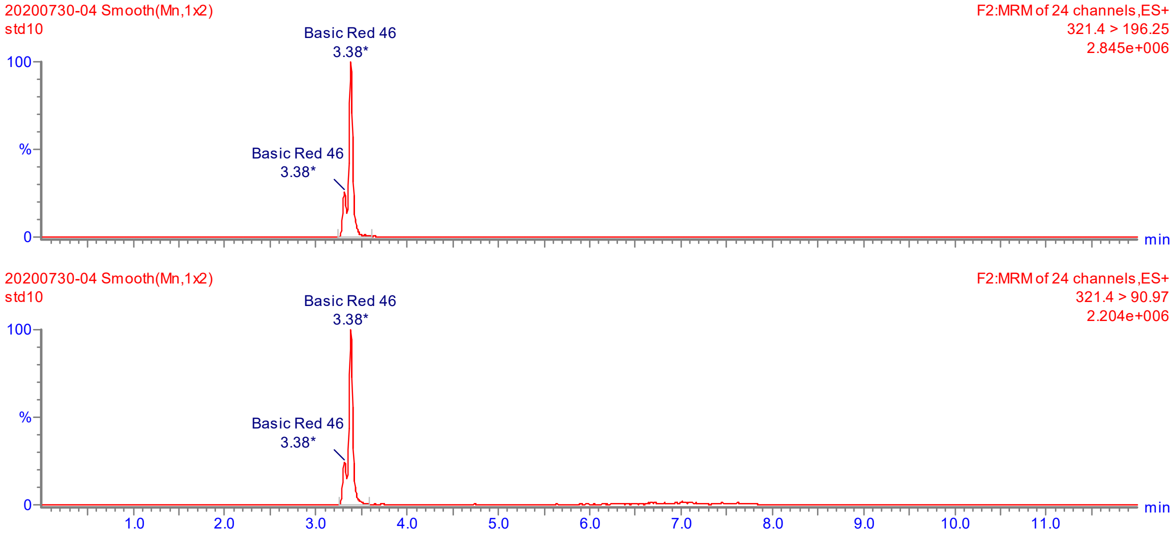
20200730-04 Smooth(Mn,1x2)
std10
Basic Red 46
3.38*
Basic Red 46
3.38*
F2:MRM of 24 channels,ES+
321.4 > 196.25
2.845e+006
100
%
0
min
20200730-04 Smooth(Mn,1x2)
std10
Basic Red 46
3.38*
Basic Red 46
3.38*
F2:MRM of 24 channels,ES+
321.4 > 90.97
2.204e+006
100
%
0
min
1.0
2.0
3.0
4.0
5.0
6.0
7.0
8.0
9.0
10.0
11.0

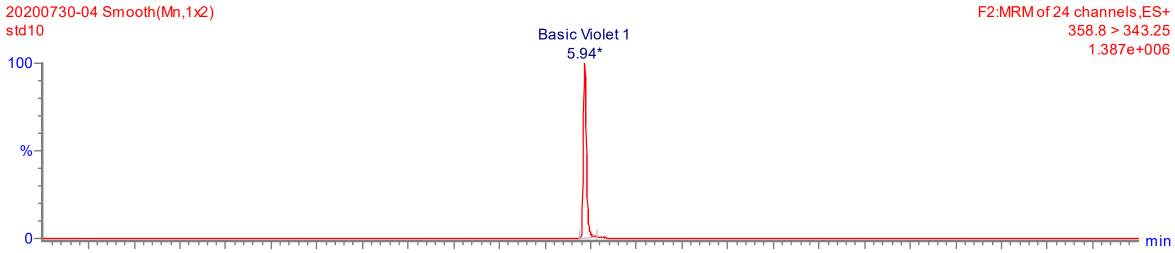
20200730-04 Smooth(Mn,1x2)
std10
Basic Violet 1
5.94*
F2:MRM of 24 channels,ES+
358.8 > 343.25
1.387e+006
100
%
0
min

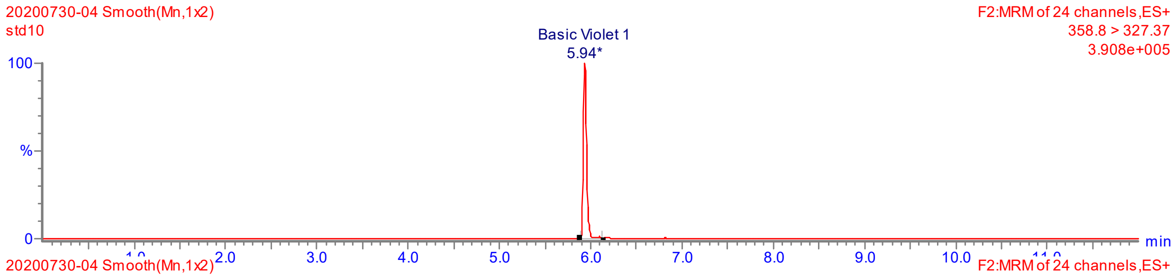
20200730-04 Smooth(Mn,1x2)
std10
Basic Violet 1
5.94*
F2:MRM of 24 channels,ES+
358.8 > 327.37
3.908e+005
100
%
0
min
2.0
3.0
4.0
5.0
6.0
7.0
8.0
9.0
10.0

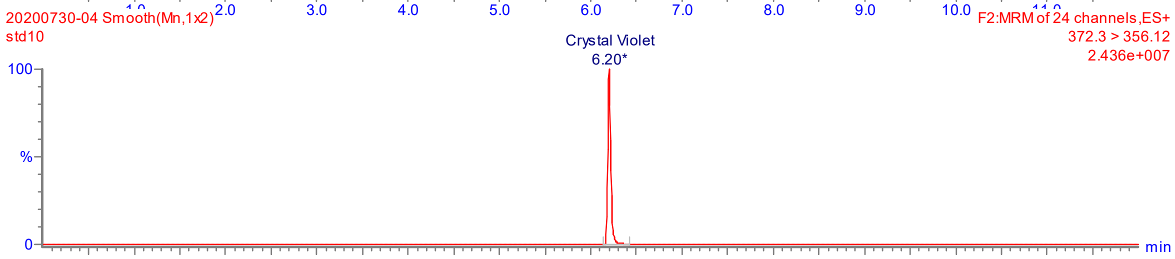
20200730-04 Smooth(Mn,1x2)
std10
Crystal Violet
6.20*
F2:MRM of 24 channels,ES+
372.3 > 356.12
2.436e+007
100
%
0
min

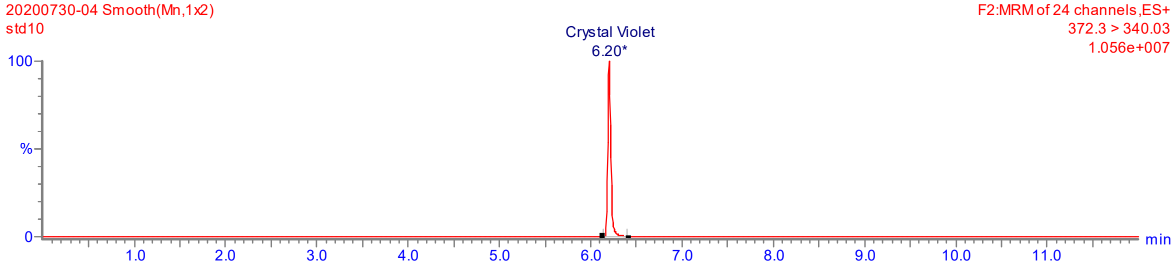
20200730-04 Smooth(Mn,1x2)
std10
Crystal Violet
6.20*
F2:MRM of 24 channels,ES+
372.3 > 340.03
1.056e+007
100
%
0
min
1.0
2.0
3.0
4.0
5.0
6.0
7.0
8.0
9.0
10.0
11.0

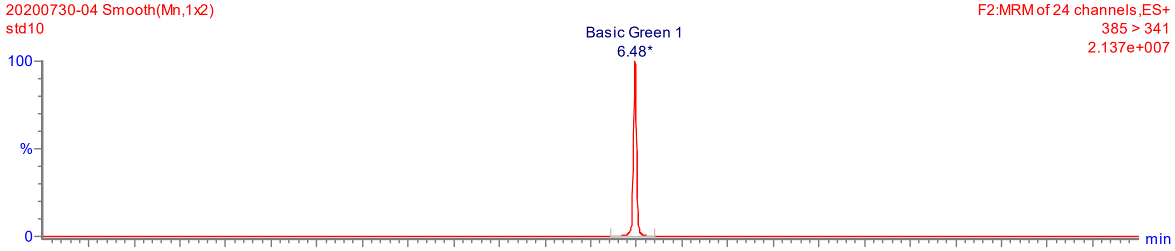
20200730-04 Smooth(Mn,1x2)
std10
Basic Green 1
6.48*
F2:MRM of 24 channels,ES+
385 > 341
2.137e+007
100
%
0
min

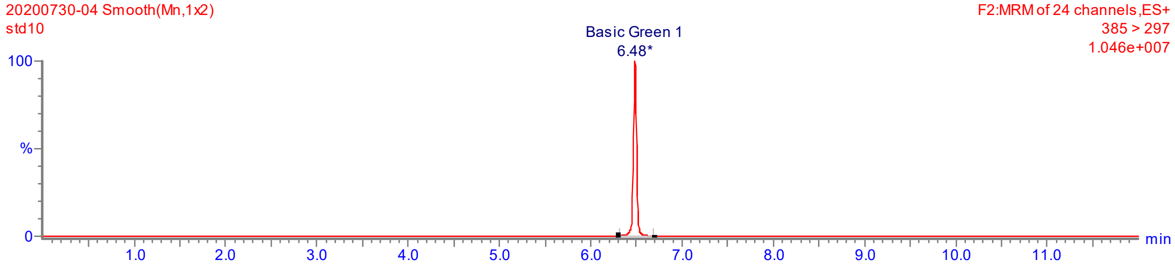
20200730-04 Smooth(Mn,1x2)
std10
Basic Green 1
6.48*
F2:MRM of 24 channels,ES+
385 > 297
1.046e+007
100
%
0
min
1.0
2.0
3.0
4.0
5.0
6.0
7.0
8.0
9.0
10.0
11.0

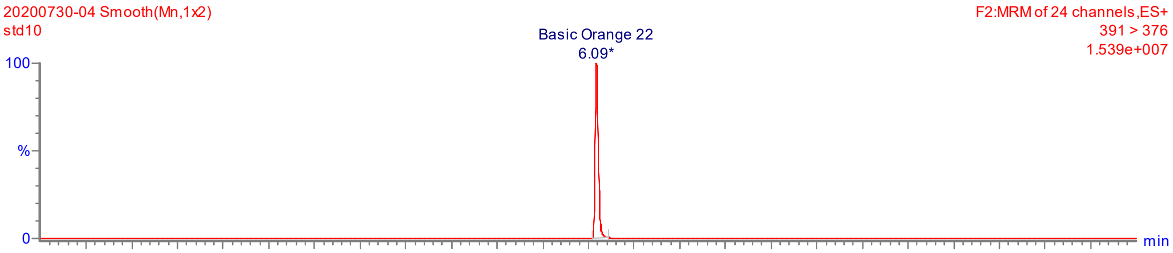
20200730-04 Smooth(Mn,1x2)
std10
Basic Orange 22
6.09*
F2:MRM of 24 channels,ES+
391 > 376
1.539e+007
100
%
0
min

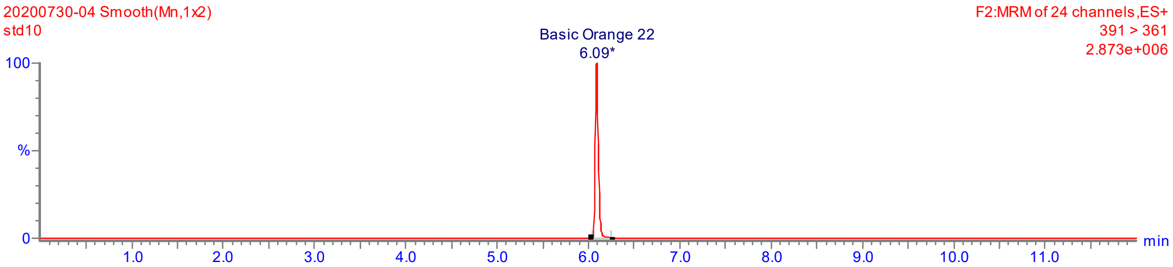
20200730-04 Smooth(Mn,1x2)
std10
Basic Orange 22
6.09*
F2:MRM of 24 channels,ES+
391 > 361
2.873e+006
100
%
0
1.0 2.0 3.0 4.0 5.0 6.0 7.0 8.0 9.0 10.0 11.0
min

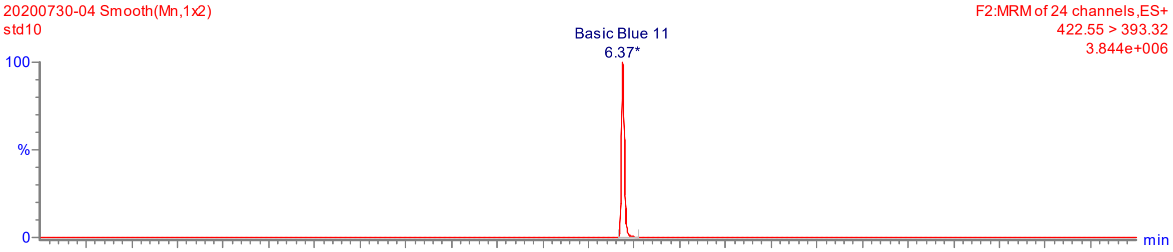
20200730-04 Smooth(Mn,1x2)
std10
Basic Blue 11
6.37*
F2:MRM of 24 channels,ES+
422.55 > 393.32
3.844e+006
100
%
0
min

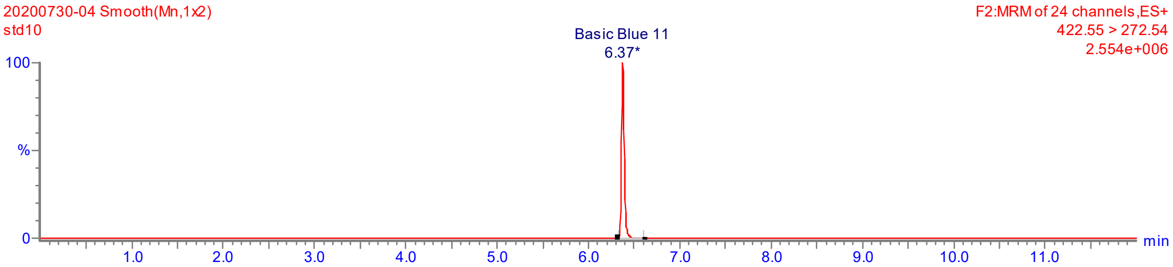
20200730-04 Smooth(Mn,1x2)
std10
Basic Blue 11
6.37*
F2:MRM of 24 channels,ES+
422.55 > 272.54
2.554e+006
100
%
0
1.0 2.0 3.0 4.0 5.0 6.0 7.0 8.0 9.0 10.0 11.0
min

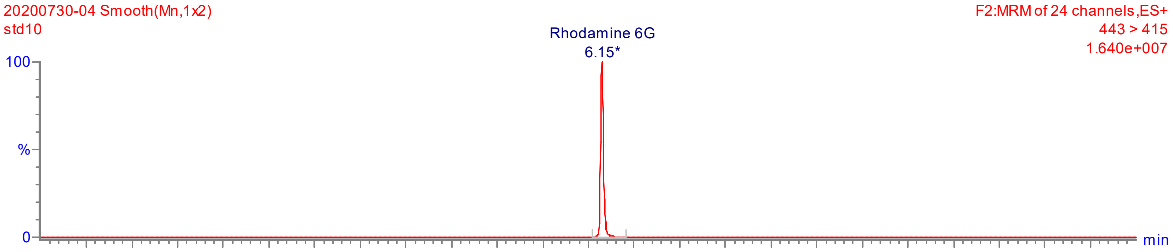
20200730-04 Smooth(Mn,1x2)
std10
Rhodamine 6G
6.15*
F2:MRM of 24 channels,ES+
443 > 415
1.640e+007
100
%
0
min

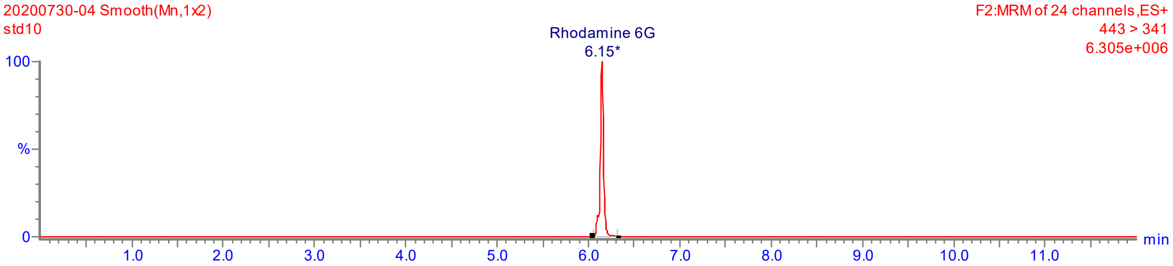
20200730-04 Smooth(Mn,1x2)
std10
Rhodamine 6G
6.15*
F2:MRM of 24 channels,ES+
443 > 341
6.305e+006
100
%
0
1.0 2.0 3.0 4.0 5.0 6.0 7.0 8.0 9.0 10.0 11.0
min

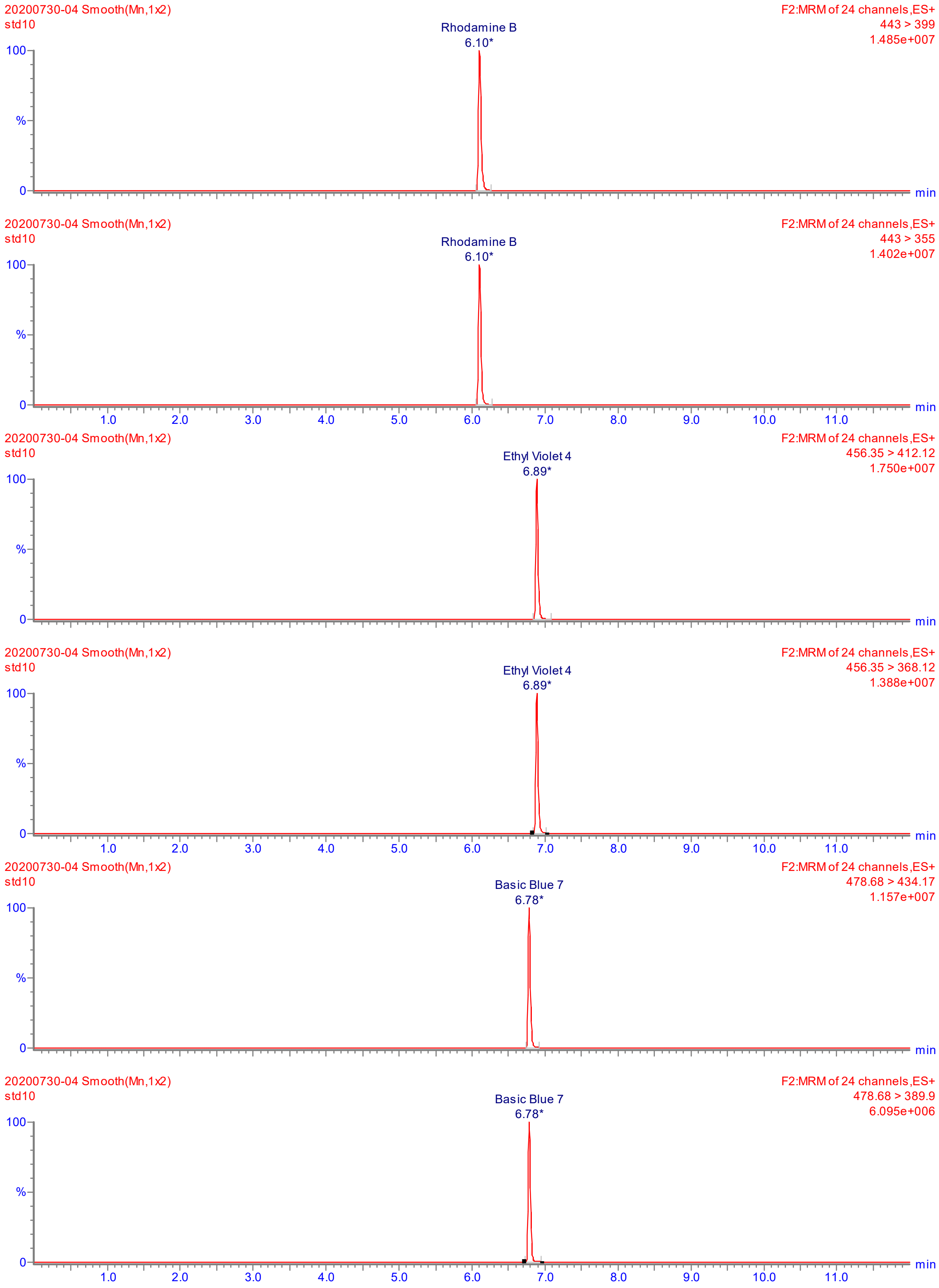
20200730-04 Smooth(Mn,1x2)
std10
F2:MRM of 24 channels,ES+
443 > 399
1.485e+007
Rhodamine B
6.10*
100
%
0
min
20200730-04 Smooth(Mn,1x2)
std10
F2:MRM of 24 channels,ES+
443 > 355
1.402e+007
Rhodamine B
6.10*
100
%
0
1.0
2.0
3.0
4.0
5.0
6.0
7.0
8.0
9.0
10.0
11.0
min
20200730-04 Smooth(Mn,1x2)
std10
F2:MRM of 24 channels,ES+
456.35 > 412.12
1.750e+007
Ethyl Violet 4
6.89*
100
%
0
min
20200730-04 Smooth(Mn,1x2)
std10
F2:MRM of 24 channels,ES+
456.35 > 368.12
1.388e+007
Ethyl Violet 4
6.89*
100
%
0
1.0
2.0
3.0
4.0
5.0
6.0
7.0
8.0
9.0
10.0
11.0
min
20200730-04 Smooth(Mn,1x2)
std10
F2:MRM of 24 channels,ES+
478.68 > 434.17
1.157e+007
Basic Blue 7
6.78*
100
%
0
min
20200730-04 Smooth(Mn,1x2)
std10
F2:MRM of 24 channels,ES+
478.68 > 389.9
6.095e+006
Basic Blue 7
6.78*
100
%
0
1.0
2.0
3.0
4.0
5.0
6.0
7.0
8.0
9.0
10.0
11.0
min

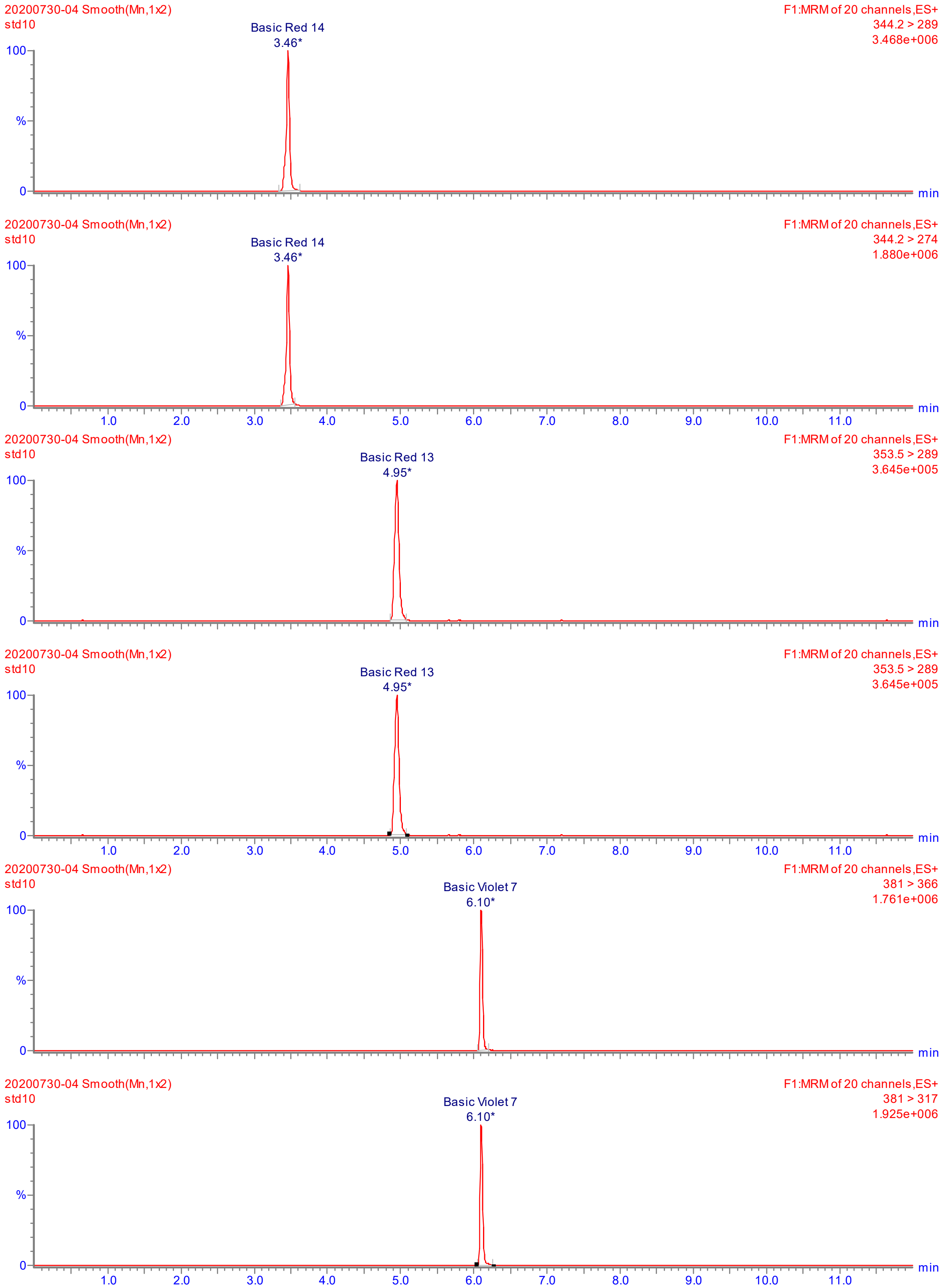
20200730-04 Smooth(Mn,1x2)
std10
Basic Red 14
3.46*
F1:MRM of 20 channels,ES+
344.2 > 289
3.468e+006
20200730-04 Smooth(Mn,1x2)
std10
Basic Red 14
3.46*
F1:MRM of 20 channels,ES+
344.2 > 274
1.880e+006
20200730-04 Smooth(Mn,1x2)
std10
Basic Red 13
4.95*
F1:MRM of 20 channels,ES+
353.5 > 289
3.645e+005
20200730-04 Smooth(Mn,1x2)
std10
Basic Red 13
4.95*
F1:MRM of 20 channels,ES+
353.5 > 289
3.645e+005
20200730-04 Smooth(Mn,1x2)
std10
Basic Violet 7
6.10*
F1:MRM of 20 channels,ES+
381 > 366
1.761e+006
20200730-04 Smooth(Mn,1x2)
std10
Basic Violet 7
6.10*
F1:MRM of 20 channels,ES+
381 > 317
1.925e+006
100
%
0
min
1.0
2.0
3.0
4.0
5.0
6.0
7.0
8.0
9.0
10.0
11.0

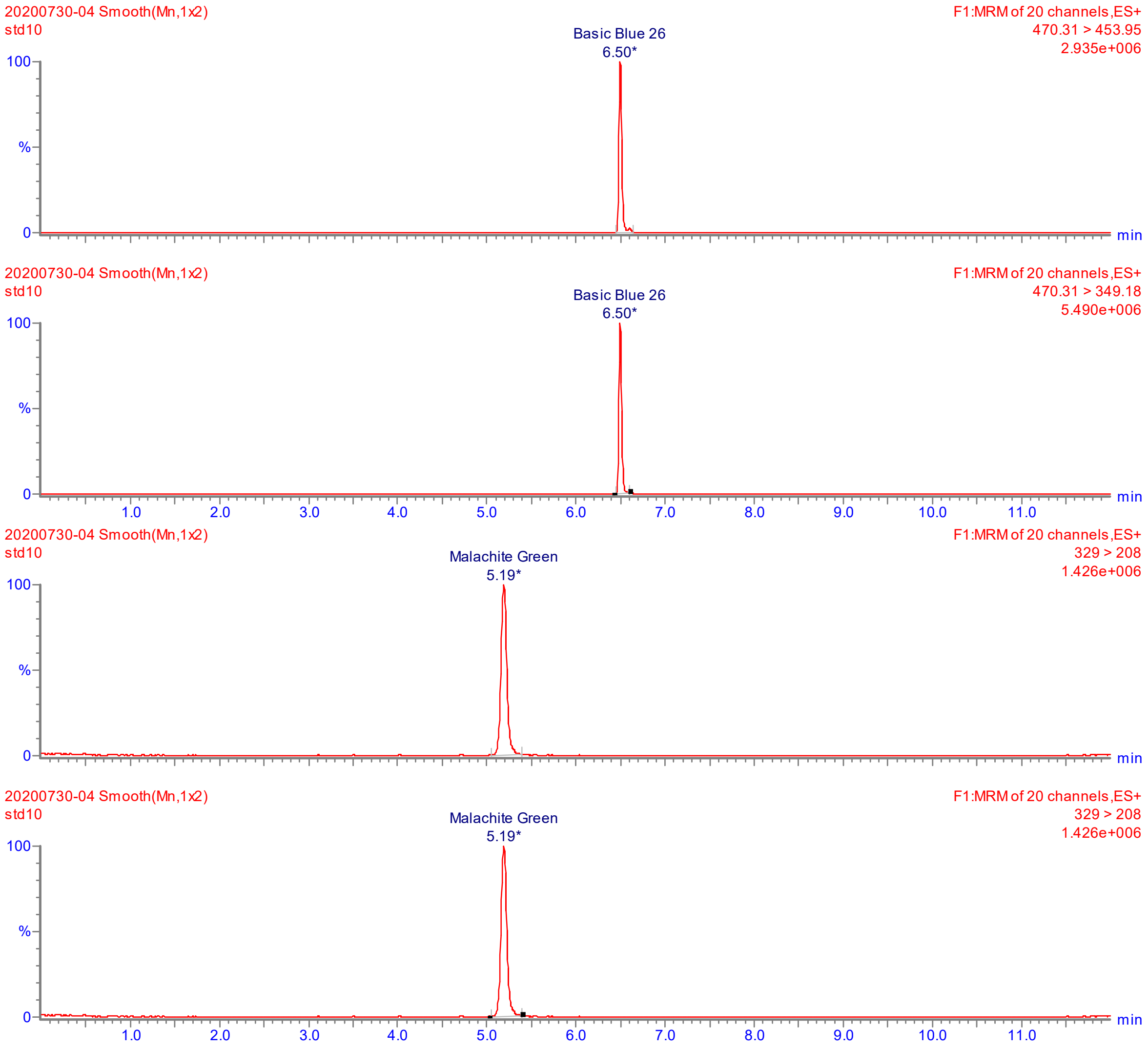

Figure S6. HPLC-MS/MS chromatograms of 16 synthetic basic dyes.
